# Supplementary figures and images for: Mistletoe Extracts Inhibit Progressive Growth of Prostate Cancer Cells
Source: Cells. 2025 Sep 30;14(19):1535. doi: 10.3390/cells14191535 (PMC12523822; doi:10.3390/cells14191535)

Figure S1, Western blots

Fig. 4B

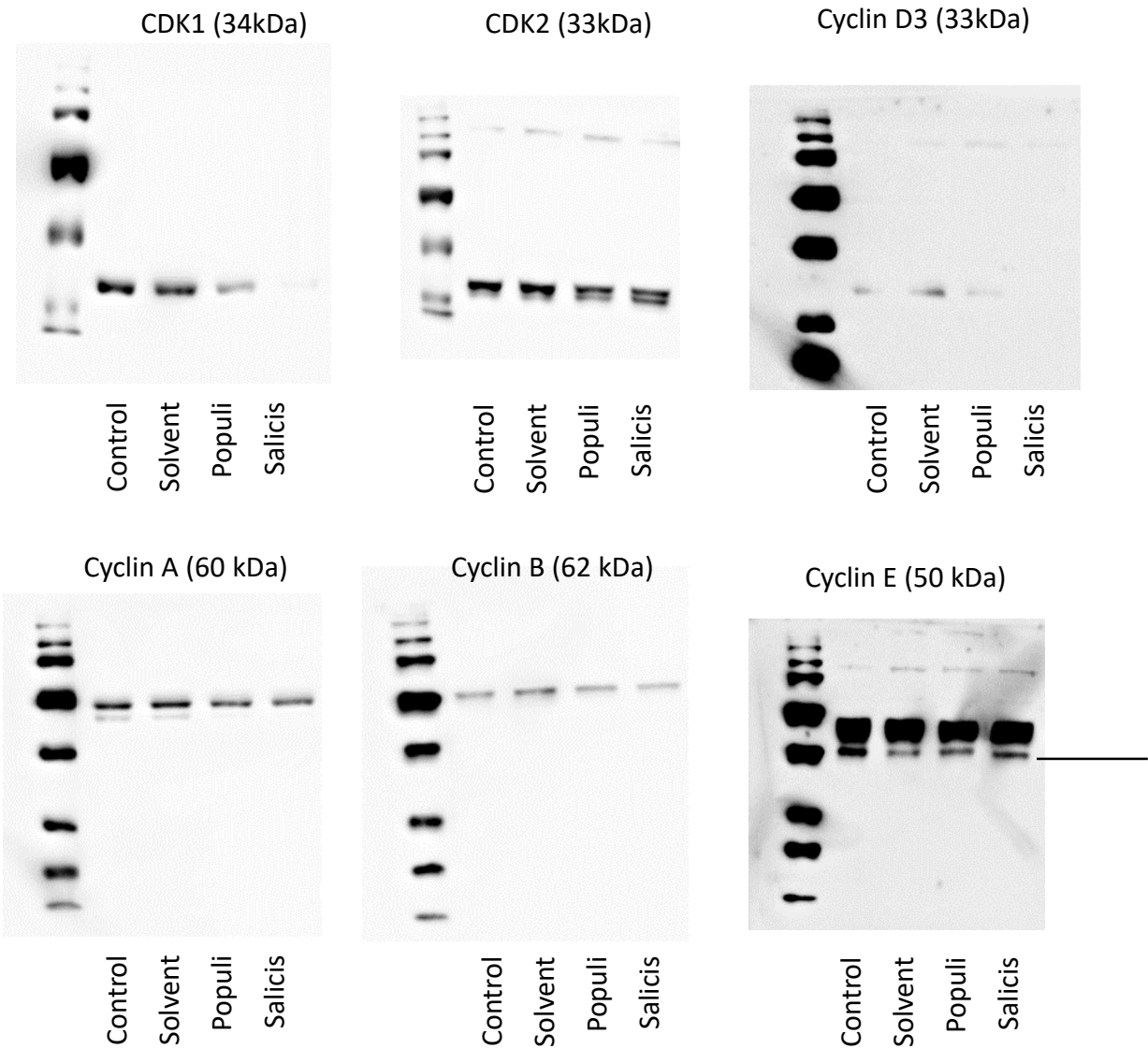

Fig. 9C

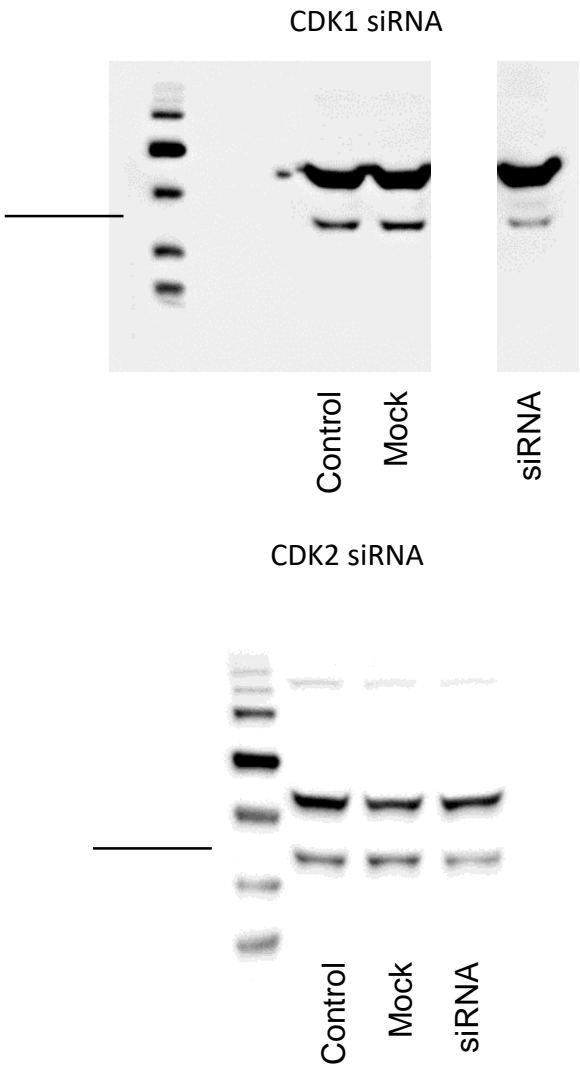

Supplement: Supplementary file 1 [file cells-14-01535-s001.zip › cells-3793932-supplementary.pdf]
